# The Overlapping Burdens of Fatigue and Daytime Sleepiness: Gender-Specific Impacts on Life Quality in Patients with Sleep Disorders

**DOI:** 10.3390/diseases13060172

**Published:** 2025-05-29

**Authors:** Bianca Temporini, Dario Bottignole, Giulia Balella, Giorgio Ughetti, Irene Pollara, Margherita Soglia, Francesco Rausa, Ylenia Ciuro, Christian Franceschini, Marcello Giuseppe Maggio, Liborio Parrino, Carlotta Mutti

**Affiliations:** 1 Sleep Disorders Center, Department of Medicine and Surgery, Parma University Hospital, A. Gramsci Street 14, 43126 Parma, Italy; 2 Neurology Unit, Department of General and Specialized Medicine, Parma University Hospital, A. Gramsci Street 14, 43126 Parma, Italy; 3 Mario Giovanni Terzano Interdepartmental Centre for Sleep Medicine, University of Parma, A. Gramsci Street 14, 43126 Parma, Italy; 4 Department of Medicine and Surgery, University of Parma, A. Gramsci Street 14, 43126 Parma, Italy; 5 Geriatric Clinic Unit, University Hospital of Parma, A. Gramsci Street 14, 43126 Parma, Italy

**Keywords:** sleep disorders, gender-related issues, psychological distress, fatigue, sleepiness

## Abstract

Background: Excessive daytime sleepiness (EDS) and fatigue are two impactful symptoms, frequently associated with sleep disorders, which can worsen the quality of life. Due to overlapping features and patient-report ambiguity a clear-cut distinction between EDS and fatigue can become a challenging issue. We aimed to investigate the prevalence and consequences of these two conditions in several sleep pathologies, examining their social, psychological, and dietary impact, with a focus on gender-related differences and occupational status. Methods: We prospectively recruited for an online survey 136 adult outpatients (60 females) affected by various sleep disorders and admitted to our Sleep Disorders Center in Parma, Italy. Patients were asked to complete the following tests: Epworth Sleepiness Scale, Fatigue Severity Scale, Pittsburgh Sleep Quality Index, Difficulties in Emotion Regulation Scale, Depression Anxiety Stress Scale-21, Hyperarousal Scale, the Addiction-like Eating Behaviors Scale, Work Productivity and Activity Impairment Questionnaire, MEDI-Lite, and EQ-5D Health Questionnaire. Results:Fatigue was the primary daily symptom leading to serious repercussions on social/emotional and psychological well-being, while daytime sleepiness showed a less relevant role. Women reported higher levels of fatigue, sleep disturbances, emotional dysregulation, hyperarousal, and work productivity impairments. Unemployed people experienced a higher degree of fatigue, with multi-level negative consequences. Conclusions: We suggest sleep clinicians place a greater emphasis on the assessment of fatigue during clinical interviews, keeping in mind the greater vulnerability of females, experiencing disproportionate consequences. Further studies should expand our findings, exploring a wider range of gender identities and recruiting larger samples of patients.

## 1. Introduction

Excessive daytime sleepiness (EDS) is described as a condition of frequent and persistent drowsiness and is typically considered a warning sign of poor sleep quality and/or underlying sleep disorders. The vast majority of scientific reports on sleep cohorts focus on EDS as one of the main daytime disturbances related to these pathologies [1].

At a clinical level, the differentiation between EDS and other similar conditions such as fatigue, clinophilia, or sleep inertia is not trivial, as patients themselves can frequently mix up terminology and interchangeably adopt different definitions with no real insight into their situation [2,3]. Fatigue is described as a condition of physical and mental weakness, partially comparable to asthenia. Both EDS and fatigue occur with high prevalence in the general population, but the overlap and similarities between the conditions yield clinical ambiguity and might favor their epidemiological underestimation [4].

In 2020, a European Task Force endorsed by the European Neurological Association (EAN), the European Sleep Research Society (ESRS), and the European Narcolepsy Network (EU-NN), while developing an updated guideline for the treatment of narcolepsy, also provided a revised terminology seeking to harmonize concepts regarding daytime sleepiness [5]. Accordingly, EDS is defined as the complaint of inability to stay awake during the normal wake period of the day, while fatigue is the complaint of physical and/or mental exhaustion with difficulties in initiating or sustaining voluntary activities that are not significantly improved by increased rest or sleep [5].

Several sleep disorders are closely linked to the development of EDS, including sleep-related breathing disorders, insomnia, and central disorders of hypersomnolence [6], while the relationship between fatigue and sleep disturbances is far less explored. Numerous studies have connected EDS and fatigue to severe physiological outcomes such as social isolation, somatic problems, and anxiety [7,8,9]. In parallel, EDS has been associated with an increased risk of cardiovascular mortality [6].

In the current study, we explored the prevalence and effects of EDS and fatigue in a cohort of adult patients affected by sleep disorders. We focused on the socio-psychological repercussions of these symptoms, hoping to determine their true clinical significance. Gender-related differences are also investigated, as well as discrepancies due to occupational status.

To thoroughly investigate symptoms of EDS and fatigue across our sample, we adopted a multidimensional approach, including not only questionnaires addressing these manifestations directly but also other instruments evaluating their linked hidden consequences. Indeed, both fatigue and sleepiness might be related to many other physical and mental manifestations, which can further impair patients’ perception of the former two studied symptoms. First of all, it is already established the presence of a mutual interplay between the individual emotional experience and regulation, and the mental or physical fatigue perception [10,11,12]. Similarly, sparse studies have highlighted the presence of an altered perception of global health status and illness itself in patients experiencing a chronic fatigue condition, with a detrimental interaction between all these factors [13,14,15]. Interestingly, chronic fatigue and its consequences on the overall quality of life may also lead to a negative effect on work productivity, with several consequences and even job loss [16,17,18] and individual employment status has been included among the most underestimated features affecting sleep health [19].

Secondly, patients chronically experiencing daytime sleepiness and fatigue have a higher burden of psychological distress, reporting mainly depressive symptoms and anxiety (particularly in those who have maladaptive perfectionism and neuroticism traits), which appeared to be proportional to symptom severity [20,21,22]. Despite the psychopathological basis underlying these mutual relations have to be fully clarified yet, an interesting element of this complex interplay is represented by hyperarousal, a well-known cognitive condition of patients affected by a chronic insomnia disorder and identified by an abnormal hyperactivation of physiological factors involved in the stress response (e.g., coping behaviors, autonomous nervous system, endocrine system) [23]. Similarly to insomniac patients, people experiencing higher levels of physical and mental fatigue display a hyperarousal state, which could explain the predisposition to developing an anxiety disorder [23,24].

Lastly, in consideration of the negative effects exerted by sleepiness and fatigue on the global subjectively perceived health and particularly on mental health, we considered it relevant to focus on the role of nutrition on these symptoms. For instance, people experiencing a chronic stressful state (such as daytime sleepiness and fatigue) are more vulnerable to eating disorders, particularly those who have specific personality traits such as impulsivity and cognitive inflexibility and could develop an addiction-like eating behavior, with a subsequent vicious circle [25]. On the other hand, some studies have previously shown the positive role of the Mediterranean diet not only on physical health but also on subjective symptoms such as fatigue [26,27].

Aware of the intricate connections between fatigue, sleepiness, and all these many facets of quality of life, we decided to shape our research with an inclusive and exploratory spirit—broadening our focus beyond the two initial symptoms to uncover their potential hidden consequences.

## 2. Materials and Methods

During a 3-month recruitment period (from September to November 2023), we prospectively enrolled the adult outpatients admitted to our Sleep Disorders Center in Parma University Hospital (Italy). Patients were invited to participate in the study at the end of their clinical visit to our Sleep Center. The great majority of patients agreed to take part, while a minority declined for various reasons (e.g., mainly lack of time, and limited interest in the proposed study). Although the final sample was limited in dimension and our initial goal was to reach up to 200 subjects, we decided to restrict the enrollment over a short (homogenous) time to reduce the possible confounders related to seasonal differences (e.g., Christmas celebrations, changes in daylight length, etc.) that can impact the test outcomes. In the end, we believe that the group that participated can be considered representative of our usual patient flow.

According to the previously mentioned transdiagnostic approach, for each patient, we collected main clinic-demographical information such as age, gender, body mass index (BMI), and working role, and we classified them according to their sleep diagnosis, as categorized in the International Classification for Sleep Disorders [28]. Accordingly, our sample included patients affected by chronic insomnia, sleep-related breathing disorders, central disorders of hypersomnolence, circadian rhythm sleep-wake disorders, parasomnia (including REM and non-REM sleep parasomnia), sleep-related movement disorders, and sleep-related epilepsy.

Furthermore, in compliance with the aforementioned complex interplays between symptoms of sleepiness and fatigue, we sought to use the following validated questionnaires in our multimodal approach to patients:

- Epworth Sleepiness Scale (ESS): it is a self-administered questionnaire with 8 questions. Respondents are asked to rate, on a 4-point scale (0–3), their usual chances of dozing off or falling asleep while engaged in eight different activities. A score above 11 is considered significant for EDS [29].
- Fatigue Severity Scale (FSS): a self-evaluating assessment establishing the impact of fatigue on daily life, including nine statements that rate the severity of related symptoms. A score above 36 is indicative of clinically significant fatigue [30].
- Pittsburgh Sleep Quality Index (PSQI): a 19 self-rated questionnaire analyzing sleep quality. It can be subdivided into 7 components measuring: subjective sleep quality, sleep latency, sleep duration, habitual sleep efficiency, sleep disturbances, use of hypnotic medications, and daytime dysfunction. The seven components yield one global score, ranging from 0 (no difficulties) to 21 (severe sleep impairment). A cut-off of 5 is considered significant for altered sleep quality [31].
- Difficulties in Emotion Regulation Scale-Short Form (DERS-SF): it is an instrument measuring emotion regulation problems, organized with 36 items self-report scale, indicative of how patients relate to their emotions, in order to produce scores on the following subscales: non-acceptance of emotional responses; difficulty engaging in goal-directed behavior, impulse control difficulties, lack of emotional awareness, limited access to emotion regulation strategies and lack of emotional clarity [32]. Higher values reflect major impairment in the explored subitem, with no specific cut-off.
- Hyperarousal Scale (H-Scale): The H-Scale consists of 26 items that assess the hyperarousal behavioral trait on a four-point Likert-type scale coded as 0 = not at all, 1 = a little, 2 = quite a bit, and 3 = extremely. The scale produces a Total Summation Score (HSUM): a score of “introspectiveness”, i.e., a possible tendency to ruminate; a score of “reactivity”; and the score of “extreme responses”, referring to the total number of items checked as “extremely” ranging from 0 to 26. The level of hyperactivation is directly related to the Total Summation Score [24].
- Depression and Anxiety Scale (DASS-21): it is a 21-item self-questionnaire measuring distress along the 3 axes of depression, anxiety, and stress. Each item is scored from 0 to 3, with higher points indicative of more severe disturbances. Final scores are divided into 3 scales, respectively analyzing depression, anxiety, and stress. Values above 21 are considered severe for depression, values above 15 are severe for anxiety, and values above 26 are severe for stress perception [33].
- Addiction-like Eating Behaviors Scale (AEBS): It represents a validated tool to quantify the behavioral features of a potential ‘eating addiction’. AEBS is based on 15 Likert questions. Values above 38 are related to compulsive eating behaviors [34].
- Work Productivity and Activity Impairment Questionnaire-General Health (WPAI-GH): It is a 6-item instrument to measure impairments over the past 7 days in both paid work and unpaid work due to one’s health. It consists of four metrics: (1) Absenteeism (the percentage of work time missed); (2) Presenteeism (the percentage of impairment experienced while at work); (3) Overall work productivity loss (an estimate of the combination of absenteeism and presenteeism), (4) Activity impairment (the percentage of impairment in daily activities) [35].
- MEDI-LITE: it measures the adherence to the Mediterranean diet, determining the consumption, in terms of daily and/or weekly quantities, of 9 food groups that are present in the scientific studies that have investigated the association between adherence to the Mediterranean diet and health status. The final score, obtained by summing these values, varies from 0 (low adherence) to 18 (high adherence) [36].
- EQ-5D-health questionnaire: it represents a quick-to-use instrument to describe individuals’ health state. The descriptive system comprises five dimensions: mobility, self-care, usual activities, pain/discomfort, and anxiety/depression. Each dimension has 5 levels: no problems, slight problems, moderate problems, severe problems, and extreme problems. Patients’ responses are coded as a number (1, 2, or 3) that corresponds to the respective level of severity: 1 indicates no problems, 2 some problems, and 3 extreme problems. In this way, a person’s health state profile can be defined by a 5-digit number, ranging from 11,111 (having no problems in any of the dimensions) to 33,333 (having extreme problems in all the dimensions) [37].

### Statistical Analysis

Statistical analyses were performed with Jamovi software, 2022 version 2.3 [38]. Descriptive analyses were conducted for all the investigated variables collected in our sample. Data normality was explored using the Shapiro-Wilk test, and alpha values below 0.05 were considered representative of a non-normal data distribution. When data were abnormally distributed, non-parametric tests were adopted for inference analyses. Conversely, if data distribution was proven to be normal, simple comparisons were carried out through an independent sample *t*-test. The strength of the relationship was explored by adopting Cohen’s effect size, expressed through the statistic d, with values considered representative of small (d = 0.2), medium (d = 0.5), and large (d = 0.8) effects, respectively.

Given the exploratory nature of this pivotal study and the preliminary hypothesis of a detrimental role for various psychometric and demographic aspects towards the development of sleepiness and fatigue, multiple comparisons were conducted across different variables using a series of ANOVA tests (or its non-parametric alternative, the Kruskal-Wallis test). According to the non-normal distribution displayed by the majority of data, when significant results were observed posthoc analyses were adopted using the Steel-Dwass-Critchlow-Fligner (SDCF) method, which performs pairwise comparisons while controlling the family-wise error rate (directly related to the type I errors probability) across all k(k − 1)/2 contrasts [39,40].

Linear regression was adopted to analyze the correlation between continuous variables. For all the inferential analyses, a *p*-value lower than 0.05 was considered significant.

## 3. Results

Our final sample included 136 patients (60 female and 76 male), slightly overweight (mean BMI 26.9 +/− 5.37), and with a mean age of 52.2 years (+/−13.4). The most prevalent sleep disorders among enrolled patients were insomnia (38.2%) and sleep-related breathing disorders (34.6%), followed by central disorders of hypersomnolence (11%), sleep-related movement disorders (5.9%), and parasomnia (5.9%). Only a minority of subjects were affected by circadian sleep-wake disorders (2.2%) or sleep-related epilepsy (2.2%). Most of the enrolled patients were currently employed (66.2%), 26.5% were retired, and 7.4% were unemployed. Furthermore, among workers, we included 21.3% of shift workers. The main results are summarized in Tables S1–S5 in Supplementary Materials.

### 3.1. Fatigue Severity Scale (FSS)

**Figure 1:**
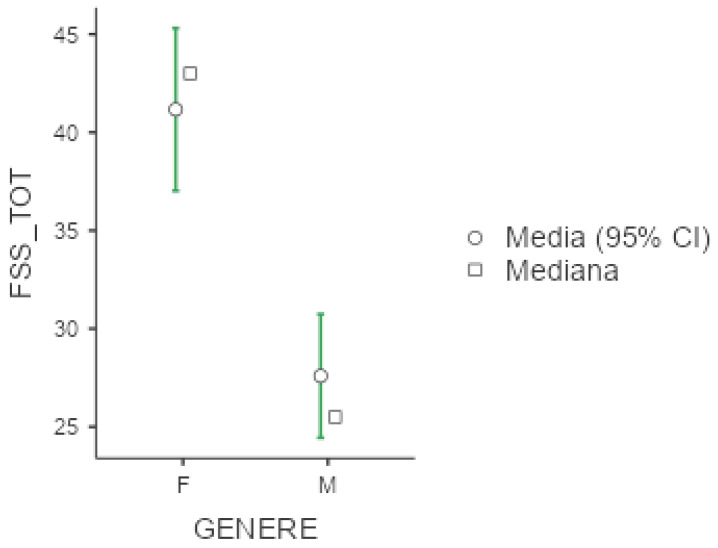
Marginal mean and median plot displaying the gender difference between males (M) and females (F) in the Fatigue Severity Scale total result (FSS_TOT). “Media”: mean; “Mediana”: median; “Genere”: gender.

The mean FSS score was 33.6 (+/−16.5), with higher scores among females (41.2 +/− 16.4, *p* < 0.001, d = 0.891), as displayed below in Figure 1. Unemployed patients reported high values (54.6 +/− 6.65) with respect to employed (*p* < 0.001) and retired individuals (*p* < 0.001). Curiously, shift workers were less affected by fatigue compared to non-shift workers (*p* = 0.045, d = 0.83), but the two groups differed in terms of age (with shift workers being significantly younger, as described in Table S4, Supplementary Materials).

Concerning sleep disorders, higher scores of FSS were detected among patients with sleep-related movement disorders (mean FSS 40.9 +/− 13.3), with no statistical differences with respect to other sleep pathologies. See Table S1 in Supplementary Materials for further details regarding the questionnaires’ results across sleep disorders.

### 3.2. Epworth Sleepiness Scale (ESS)

**Figure 2:**
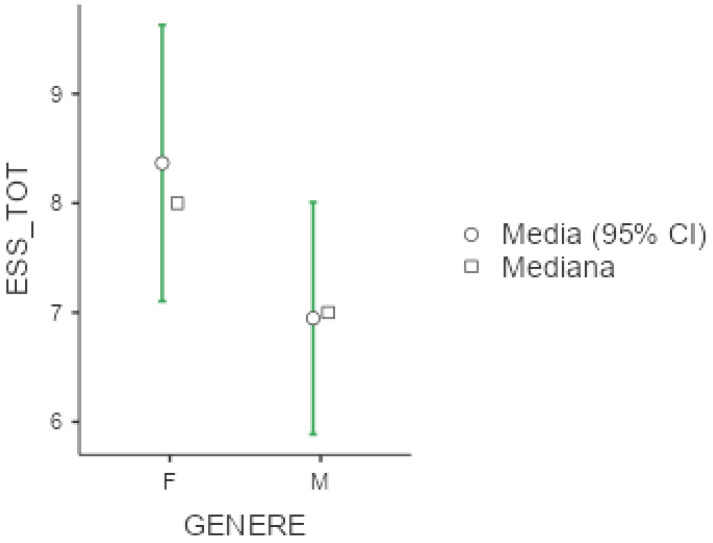
Marginal mean and median plot displaying the gender difference between males (M) and females (F) in the Epworth Sleepiness Scale result (ESS_TOT). “Media”: mean; “Mediana”: median; “Genere”: gender.

Mean ESS was 7.57 (+/−4.88), with slightly higher scores among females (8.37 +/− 4.99) versus males (6.95 +/− 4.72), as shown below in Figure 2. No significant differences emerged with respect to occupational status or shift working habits, although we noticed that unemployed workers and non-shift workers presented higher values at ESS (respectively 8.10 +/− 5.69 and 7.64 +/− 4.78). With respect to sleep disorders, as expected, patients with central disorders of hypersomnolence presented the highest ESS scores (11.9 +/− 4.67), followed by patients with sleep-related movement disorders.

### 3.3. Pittsburgh Sleep Quality Index (PSQI)

**Figure 3:**
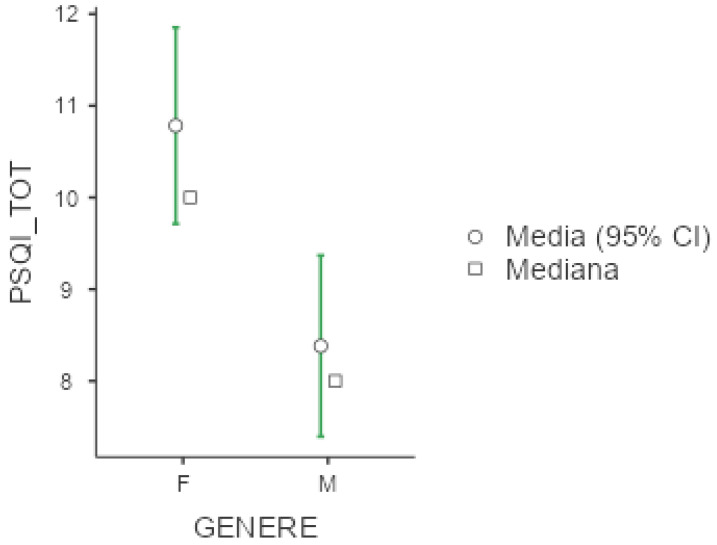
Marginal mean and median plot displaying the gender difference between males (M) and females (F) in the Pittsburgh Sleep Quality Index total score (PSQI_TOT). “Media”: mean; “Mediana”: median; “Genere”: gender.

Mean PSQI was 9.44 +/− 4.47, again with higher values among females (10.8 +/− 4.23) compare to men (8.38 +/− 4.39; *p* = 0.002, d = 0.55), as displayed below in Figure 3. Exploring PSQI subitems, women presented higher scores also in terms of subjectively perceived sleep quality (*p* = 0.004, d = 0.50), sleep latency (*p* = 0.013, d = 0.44), and sleep efficacy (*p* = 0.036, d = 0.36). Unemployed workers were more vulnerable in terms of overall sleep quality as measured by PSQI (*p* = 0.025, ε = 0.054), subjectively perceived sleep quality (*p* = 0.043, ε = 0.047), and daily disturbances (*p* = 0.004, ε = 0.080). Shift workers presented higher scores in the subitem of PSQI exploring sleep duration (*p* = 0.038, d= −0.454).

With regard to sleep disturbances, patients affected by insomnia complained higher values in the PSQI subitem exploring sleep quality (*p* = 0.004, ε = 0.143), while patients with sleep-related epilepsy presented higher scores in the subitem of the questionnaire measuring the utilization of hypnotic drugs (*p* < 0.001, ε = 0.223) and patients with sleep-related movement disorders presented the overall higher scores at the PSQI (*p* = 0.001, ε = 0.136). Post-hoc analysis confirmed significant differences between patients affected by insomnia compared to subjects with sleep-related breathing disorders in terms of global PSQI score (*p* = 0.001), subjectively perceived sleep quality (*p* = 0.004), and utilization of hypnotic drugs (*p* ≤ 0.001).

### 3.4. Difficulties in Emotion Regulation Scale—Short Form (DERS-SF)

Mean DERS-SF score was 41 (+/−11.5), with higher values in females (44.2 +/− 12.2) compare to men (38.5 +/− 10.4; *p* = 0.004, d = 0.51). Women also presented higher scores in the subitem of the questionnaire exploring the lack of emotional awareness (*p* = 0.44, d = −0.35), difficulty in engaging in goal-directed behavior (*p* = 0.005, d = 0.50), and limited access to emotion regulation strategies (*p* = 0.004, d = 0.51). Unemployed patients presented significantly higher values in the same subitems affected in women: analyzing the lack of emotional awareness (*p* = 0.010, ε = 0.068), difficulty in engaging in goal-directed behavior (*p* = 0.049, ε = 0.044), and limited access to emotion regulation strategies (*p* = 0.006, ε = 0.076). No significant differences emerge with respect to shift-working habits.

In terms of sleep disorders, patients with circadian sleep-wake disorders presented a higher score in the DERS-SF subitem exploring the lack of emotional awareness (*p* = 0.003, ε = 0.146). Post-hoc analysis identified significant differences between patients affected by insomnia and patients with sleep-related breathing disorders, to the disadvantage of insomniacs.

### 3.5. Addiction-like Eating Behavior Scale (AEBS)

The mean AEBS score was 38.3 (+/−7.64), indicative of high risk for eating-compulsive behaviors, with no significant gender-related differences, occupational status, and/or shift-working habits.

### 3.6. Hyperarousal Scale (H-Scale)

Mean H-Scale score was 26.5 (+/−13.7), with higher values among females (32.5 +/− 15.3) compared to males (21.8 +/− 10.2, *p* < 0.001, d = 0.82). Women presented higher values in all the subitems of the scale, respectively exploring introspection (*p* = 0.001, d = 0.57), reactivity (*p* < 0.001, d = 0.67), and extreme responses (*p* < 0.001, d = 0.82). No differences were noticed concerning employment status or shift-working habits. With respect to sleep disturbances, patients affected by insomnia presented significantly higher scores in the test (*p* = 0.033, ε= 0.102) and in the subitem analyzing introspection (*p* = 0.24, ε = 0.102). Post-hoc analysis revealed significant differences between patients with insomnia and those with sleep-breathing disorders (*p* = 0.023), for the global score of the H-scale, to the disadvantage of insomniac.

### 3.7. Work Productivity and Activity Impairment Questionnaire-General Health (WPAI-GH)

The mean percentage of work time missed due to health problems in the last 7 days was estimated at around 5%, with higher values among females (8%) compared to men (3%). The percentage of impairment experienced while at work was 32% (40% among females and 26% among males). Overall work productivity loss was 32.4% (42% in females and 26% in males), and finally, the percentage of impairment in daily activities was 38.7% (47% in females and 31% in males). Shift workers were not significantly different from non-shift workers. Lastly, no significant differences emerge with respect to sleep disorders diagnosis.

### 3.8. EQ-5D Health Questionnaire

With respect to the dimensions explored by this questionnaire, we collected the following results: mean score for mobility was 1.54 (+/−0.82), self-care 1.18 (+/−0.50), usual activities 1.67 (+/−0.93), pain/discomfort 2.26 (+/−0.96) and anxiety/depression 2.33 (+/−0.97). The general health status as described by the test was 3.73 (+/−2.37). Women presented higher scores in the subitems exploring movement (*p* = 0.040, d = 0.36), usual activities (*p* < 0.001, d = 0.62), pain/discomfort (*p* < 0.001, d = 0.70), anxiety/depression (*p* < 0.001, d = 0.69) and global health status (*p* < 0.001, d = 0.65).

Unemployed patients presented higher scores in the subitems exploring movement (*p* = 0.012, ε = 0.065), self-care (*p* = 0.022, ε = 0.056), and usual activities (*p* = 0.002, ε = 0.089) compared to employed and retired subjects. No significant differences were revealed either between shift and non-shift workers or depending on sleep diagnosis.

### 3.9. Depression and Anxiety Scale (DASS-21)

**Figure 4:**
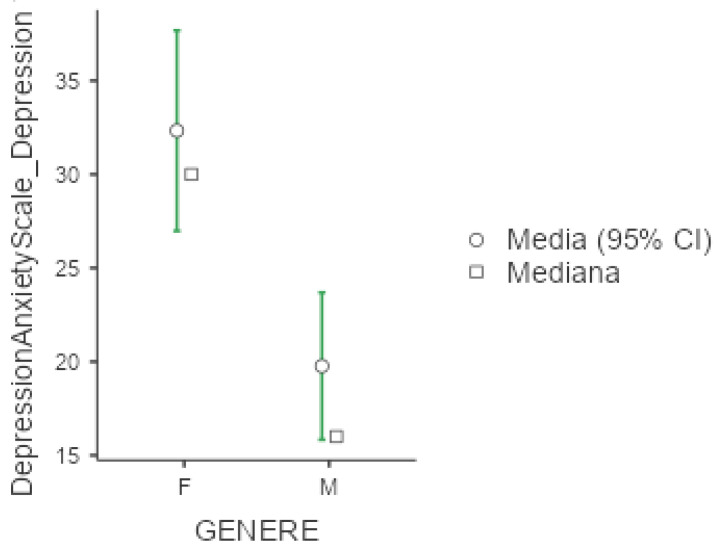
Marginal mean and median plot displaying the gender difference between males (M) and females (F) in the Depression and Anxiety Scale subitem “Depression” score. “Media”: mean; “Mediana”: median; “Genere”: gender.

**Figure 5:**
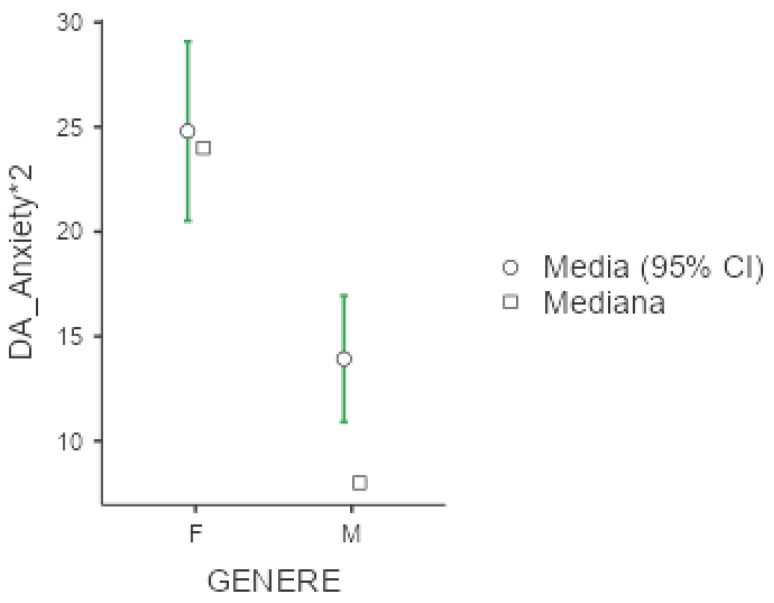
Marginal mean and median plot displaying the gender difference between males (M) and females (F) in the Depression and Anxiety Scale subitem “Anxiety*2” score. “Media”: mean; “Mediana”: median; “Genere”: gender.

**Figure 6:**
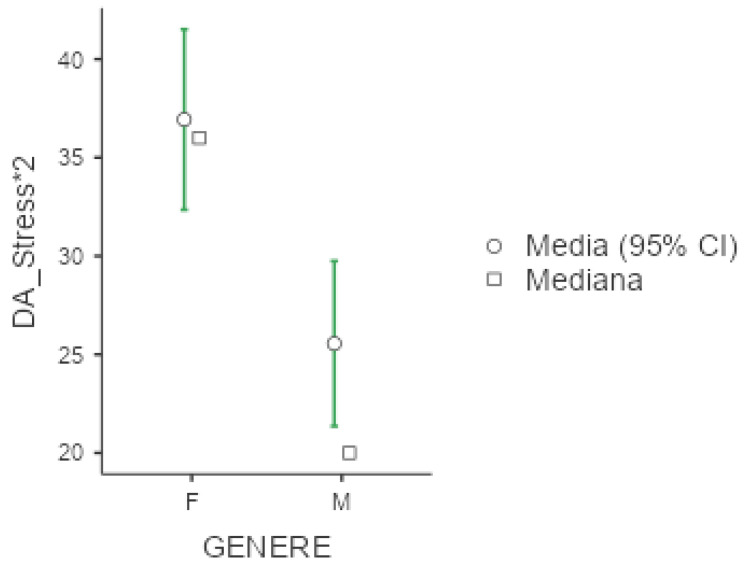
Marginal mean and median plot displaying the gender difference between males (M) and females (F) in the Depression and Anxiety Scale subitem “Stress*2” score. “Media”: mean; “Mediana”: median; “Genere”: gender.

Mean value of DASS-21 were 25.3 (+/−20.1) for depression, 18.7 (+/−16) for anxiety and 30.6 (+/−19.2) for stress. In all three subgroups explored by this test, females presented significantly higher values compared to men (*p* < 0.001 for the three comparisons), as presented below in Figure 4, Figure 5 and Figure 6. No significant differences were obtained with respect to occupational status or shift-working habits. Regarding sleep disorders, the ANOVA revealed significant differences in patients affected by circadian sleep-wake disorder in the anxiety dimension (*p* = 0.003, ε = 0.150). The post-hoc analysis documented significant differences between insomniac and sleep-disorders patients and between insomniac and hypersomnolent patients in terms of anxiety (respectively *p* = 0.014 and *p* = 0.027).

### 3.10. MEDILITE

The mean score of MEDILITE was 8.67 (+/−2.64), with higher values, indicative of adherence to the Mediterranean diet, among women (*p* = 0.037, d = 0.36), while shift workers were characterized by lower adherence to this alimentary regime (*p* = 0.023, d = 0.45). No significant difference emerged with regard to occupational status or sleep disorder diagnoses.

### 3.11. Impact of ESS and FSS on Questionnaire Results

We therefore explored whether daytime sleepiness or fatigue might impact questionnaires’ responses. FSS was strongly associated with most of the dimensions explored, correlating with PSQI, DERS-SF, AES, H-scale, WPAI-GH, EQ-5D, and DASS-21 questionnaires’ results, with relevant associations in numerous subitems of the listed questionnaires, as detailed in Table S5 (Supplementary Materials). Conversely, ESS was less strongly associated with the main questionnaires’ results, with regression analysis documenting a significant relationship with only a few subitems of PSQI (sleep latency *p* = 0.008; sleep efficacy *p* = 0.039, sleep disturbances *p* = 0.001; daytime disturbances *p* < 0.001) and WPAI-GH (see Table S5 in Supplementary Materials—for further details).

## 4. Discussion

Using a trans-diagnostic approach, we found that fatigue had a higher effect than daytime sleepiness on a variety of psychological, social, and health-related variables, with notable gender variations. Furthermore, we documented a significant susceptibility among unemployed workers if compared to employed and retired individuals, and we revealed few frailties among shift workers, especially one of the dimensions explored with PSQI (sleep duration *p* = 0.038) and MEDI-LITE (*p* = 0.023).

In the comparison across sleep disorders, the most impactful consequences were observed in the subgroup of patients with insomnia and circadian sleep-wake disorders, especially regarding the psychological consequences of the diseases. In particular, the post-hoc Steel-Dwass-Critchlow-Fligner pairwise ranking nonparametric method attested that patients affected by chronic insomnia reported a lowered sleep quality (*p* = 0.004) and a higher utilization of hypnotic drugs (*p* < 0.001) compared to subjects with sleep-related breathing disorders.

Currently, scientific literature focuses mostly on EDS while exploring sleep pathologies. Specifically, EDS has been associated with the development of depression and high levels of perceived stress [41,42,43], with augmented social and economic burden [44], and lowered quality of life [45].

In parallel, fatigue has been largely evaluated among patients affected by systemic disorders, including liver diseases [46], rheumatological disorders [47], or hematological pathologies [48]. Numerous studies have also linked fatigue to neurological disorders such as Multiple Sclerosis (MS) [49] or Parkinson’s disease [50], with only a minority of studies exploring the association between sleep disorders and fatigue [51].

According to our results fatigue significantly impacts patients’ emotional vulnerability, leading to a lack of emotional awareness (*p* ≤ 0.001), difficulty in engaging in goal-directed behavior (*p* ≤ 0.001), impulse control difficulties (*p* = 0.025), limited access to emotion regulation (*p* ≤ 0.001), depression (*p* ≤ 0.001), anxiety (*p* ≤ 0.001), stress (*p* ≤ 0.001), eating compulsive behavior (*p* < 0.001), hyperarousal (*p* = 0.001) and quality of life (*p* < 0.001). Conversely, EDS did not influence most of the explored questionnaires, except for work-related impairment due to health, as detailed in Table S5 (Supplementary Materials).

Fatigue has been associated with altered connectivity profiles in the cortico-thalamic-basal-ganglial and cortico-limbic networks [52]. Brain imaging structural findings (to note, mostly collected from MS research) suggest a key role for the thalamus in fatigue development [53,54]. The thalamus exerts a central role both as a relay station in multisensorial pathways’ regulation and as a modulatory area for consciousness and alertness, hence, it is not surprising that its abnormal functioning might be involved in fatigue development. Cortical brain areas (frontal, parietal, and temporal cortices) and components of the limbic system (cingulum and cingulate gyrus), involved in complex functions moving from motivational processes, and emotion regulation to higher cognitive functions, are also associated with this disturbance [52].

Currently, there are only a few subjective methods to assess fatigue, but no current gold standard has been established. FSS is among the most widely used measures for fatigue assessment, and it considers the disturbance on one dimension. Conversely, other questionnaires subdivided the condition into distinct dimensions, such as physical fatigue and cognitive one [55].

A comprehensive evaluation of fatigue among patients affected by sleep disorders, with a proper characterization of all its nuances, it’s advisable.

Another relevant result of our study is related to the striking gender-related differences, with women experiencing a higher burden of all the main psychological and social domains.

It is known that women have double the risk for depression and post-traumatic stress disorder (PTSD) than men, with severe autonomic and cardiovascular consequences [56]. Women live longer than men, although the longer life expectancy enjoyed by women is not directly defined by additional healthy years of life. This phenomenon is described as the “male-female health-survival” paradox, and relies on biological, psychological, and genetic factors, immune system responses, hormones, and disease patterns, but also on the persistence of work and social discrepancies that expose women to a high level of susceptibility [57]. According to the World Health Organization, in Italy, life expectancy at birth is around 84 years for women and 80 years for male, while healthy life expectancy is estimated to be around 71.1 years and 70 years respectively for women and men, hence women have to face, on average, 13 years of unhealthy life compare to the 10 involving men [58]. The women’s excess unhealthy life years have been related to a higher disability burden due to musculoskeletal diseases and anxiety-depression, the latter largely influenced by sleep and sleep disturbances [59]. Female sex increases the clinical-psychological burden also towards dramatic scenarios, as recently occurred during the SARS-CoV-2 pandemic [60].

Given the role of sleep and stress as crucial determinants of health and quality of life, it remains critical to sustain medical approaches sensitive to these topics, and probably, instead of speaking on mere longevity, we should always consider healthy aging as a goal.

In the present study, we limited our observation to male and female, although we are aware of the existence of other important categories (e.g., transgender, agender, or non-cisgender), that probably warrant dedicated attention to reliably assess eventual sleep-related consequences. Further studies involving wider samples and inclusive of all these groups would be necessary to truly understand the complexity of the multifaceted gender-related differences.

Although limited by the reduced number of enrolled patients, some interesting differences were revealed with respect to sleep-disorder categories.

As expected, patients with chronic insomnia presented higher scores in the H-scale, especially in the subitem evaluating introspection. Hyperarousal is an abnormal state of increased responsiveness to external and internal stimuli associated with increased levels of alertness and anxiety, with a large impact on sleep quantity and quality [61]. The condition has been associated with dysregulation of the hypothalamic-pituitary-adrenal axis, over-expression of the orexinergic pathway, and a down-regulation of the adenosine and serotonin circuits [61]. These observations remarked that chronic insomnia should not be considered a patients’ misperception, as it may be the result of a dissociation between arousal and sleep-inducing brain systems, elevating the condition to a real pathology.

We observed that epileptic patients largely employed hypnotic drugs, as confirmed by the PSQI subitem analyzing the use of medication, probably reflecting the tendency for over-medicalization by the clinicians who chose to adopt pharmacological over non-pharmacologic approaches, rather than patients themselves needing the comfort of molecular control over their sleep-related issues.

Major psychological consequences were noticed among patients affected by chronic insomnia and circadian sleep-wake disorders. The misalignment of circadian clocks is known to amplify the risk for severe psychiatric consequences, and the circadian rhythm is included in the determination of cardiovascular health [62].

Unemployed workers complained lowered sleep quality (*p* = 0.025, ε^2^ = 0.054), lack of emotional awareness (*p* = 0.010, ε^2^ = 0.068), difficulty in engaging in goal-directed behavior (*p* = 0.049, ε^2^ = 0.0447), limited access to emotion regulation (*p* = 0.006, ε^2^ = 0.0764), daytime impairment due to health (*p* = 0.017, ε^2^ = 0.060) with specific impact in the dimensions exploring movement (*p* = 0.012, ε^2^ = 0.065), self-care (*p* = 0.022 ε^2^ = 0.056) and daytime activities (*p* = 0.002 ε^2^ = 0.089) when compared to employed and retired patients.

The role of fatigue in the context of unemployment and job search has already been observed in previous studies [63]. Accordingly, financial difficulties and social exclusion can lead to job search fatigue, which in turn lowers organizational commitment and increases the risk of burnout. Wide-sample data collected from US and European registers based on a telephone survey conducted by the Center for Disease Control (CDC) regarding healthy-risk behaviors, chronic health conditions, and use of preventive services revealed that unemployment strongly affects sleep quality and duration [64]. Our sample confirms this detrimental relationship between joblessness and sleep disruption.

In our survey, shift workers were not particularly disadvantaged compared to non-shift workers, with the only exception of lowered sleep duration, as measured through PSQI (*p* = 0.038, d= −0.454) and the DERS-SF subitem analyzing the non-acceptance of emotional responses (*p* = 0.045, d= −0.4265). A significant difference in terms of age is to be considered, as shift workers were younger than non-shift workers. Surprisingly, fatigue perception was higher among non-shift workers, while the two groups were similar in terms of daytime sleepiness. Finally, among shift workers, the adherence to the Mediterranean diet was significantly lower (*p* = 0.023, d = 0.45). Shift-working has been associated with an increased overall risk of adverse mental health specifically for depressive symptoms, with a stronger impact among females [65]. Lower sleep duration has been proven to increase cardiovascular burden [66], and the available evidence suggests that a sleep duration of 7–8 h per day is the one most favorably associated with health among adults and older adults [67]. Given that around 1 in 5 people in Europe is a shift worker, the workplace health promotion policies should support employees’ health with a multi-domain intervention, such as frequent assessments and educational programs, including sleep hygiene and recommendations for adequate sleep duration.

Interestingly, our survey documented an overall low adherence to the Mediterranean diet among patients affected by sleep disorders, with MEDI-LITE score ranging from 7 (circadian rhythm sleep-wake disorders, CRSWD) to 9.88 (parasomnia patients), and a tendency for eating compulsive behaviors among patients affected by insomnia, sleep-related breathing disorders, circadian sleep-wake disorders, and sleep-related movement disorders. Lower scores at AES were obtained by patients suffering from parasomnia, sleep-related epilepsy, and hypersomnolence disorders. Diet and eating behaviors are among the major determinants of human health, with a tight association with sleep and sleep quality. Adherence to the Mediterranean diet has been proven to ameliorate sleep satisfaction and duration among patients with chronic insomnia [68]. The association of dietary characteristics with other sleep-related pathologies is far less explored.

Our study presents some limitations: to prevent the confounding influence of seasonal changes and upcoming celebrations, we confined enrollment to a short period of time (3 months), resulting in a sample size of 136 patients. Furthermore, although we prospectively enrolled all the incoming patients over a 3-month period of time, a few patients did not agree to take part in our study and thus a potential selection cannot be completely ruled out. Also, some sleep disorders, by chance, are less represented (e.g., sleep epilepsies) than others (e.g., insomnia). Even though this partially reflects the different epidemiology of these pathologies, it can also affect our results, limiting their generalization. Further studies with wider cohorts are advisable to confirm our preliminary observations and to permit some other sub-categorization among sleep-disordered patients (e.g., mild versus severe OSA). Nevertheless, we believe that our main results, especially the central contribution of fatigue over daytime sleepiness in the determination of major socio-psychological consequences of sleep disorders, and the strong vulnerability observed among females, can be considered significant in the field of sleep research.

## 5. Conclusions

Our study emphasizes the strong impact of fatigue over EDS on various dimensions of life among adult patients with sleep disorders. Fatigue, rather than EDS, appears as the key symptom influencing social, emotional, and psychological well-being in our sample. Women, regardless of their sleep diagnosis, reported the highest level of fatigue, with greater psychological impairment and emotional perturbation compared to men, suggesting the necessity for gender-sensitive approaches in the clinical management of sleep pathologies. Important frailties were also detected among unemployed patients, presenting a high degree of fatigue with several daytime-related impairments, and probably needing tailored intervention.

Among sleep-disordered patients, the most vulnerable categories are represented by those affected by chronic insomnia and circadian sleep-wake disorders, facing the most severe socio-psychological consequences.

The presented results highlight the need for a nuanced understanding of fatigue and EDS in sleep disorders, inclusive of gender differences.

## Data Availability

The original contributions presented in this study are included in the article/Supplementary Materials. Further inquiries can be directed to the corresponding authors.

## Supplementary Materials

The following supporting information can be downloaded at: https://www.mdpi.com/article/10.3390/diseases13060172/s1, Table S1: “Questionnaires results according to sleep disorder diagnosis”; Table S2: “Questionnaires results according to gender-related differences”; Table S3: “Questionnaires results according to occupational status”; Table S4: “Questionnaires results according to shift-working”; Table S5: “Correlation analysis between FSS and ESS and main questionnaires’ results”.

## Abbreviations

The following abbreviations are used in this manuscript:

EDS	Excessive Daytime Sleepiness
EAN	European Academy of Neurology
ESRS	European Sleep Research Society
EU-NN	European Narcolepsy Network
BMI	Body Mass Index
ESS	Epworth Sleepiness Scale
FSS	Fatigue Severity Scale
PSQI	Pittsburgh Sleep Quality Index
DERS-SF	Difficulties in Emotion Regulation Scale—Short Form
DASS-21	Depression and Anxiety Scale
AEBS	Addiction-like Eating Behaviors Scale
WPAI-GH	Work Productivity and Activity Impairment Questionnaire-General Health
CRSWD	Circadian Rhythm Sleep-Wake Disorders
OSA	Obstructive Sleep Apnea

## References

[1] Guilleminault C., Brooks S.N. (2001). Excessive daytime sleepiness: A challenge for the practising neurologist. Brain.

[2] Shen J., Barbera J., Shapiro C.M. (2006). Distinguishing sleepiness and fatigue: Focus on definition and measurement. Sleep Med. Rev..

[3] Shahid A., Shen J., Shapiro C.M. (2010). Measurements of sleepiness and fatigue. J. Psychosom. Res..

[4] Pigeon W.R., Sateia M.J., Ferguson R.J. (2003). Distinguishing between excessive daytime sleepiness and fatigue. J. Psychosom. Res..

[5] Lammers G.J., Bassetti C.L., Dolenc-Groselj L., Jennum P.J., Kallweit U., Khatami R., Lecendreux M., Manconi M., Mayer G., Partinen M. (2020). Diagnosis of central disorders of hypersomnolence: A reappraisal by European experts. Sleep Med. Rev..

[6] Bock J., Covassin N., Somers V. (2022). Excessive daytime sleepiness: An emerging marker of cardiovascular risk. Heart.

[7] Jennum P.J., Plazzi G., Silvani A., Surkin L.A., Dauvilliers Y. (2021). Cardiovascular disorders in narcolepsy: Review of associations and determinants. Sleep Med. Rev..

[8] Hasler G., Buysse D.J., Gamma A., Ajdacic V., Eich D., Rössler W., Angst J. (2005). Excessive Daytime Sleepiness in Young Adults. J. Clin. Psychiatry.

[9] Sameer H.M., Imran N., Tarar T.N., Khawaja I.S. (2020). Association of Excessive Daytime Sleepiness With Psychological Distress in Medical Students. Prim Care Companion CNS Disord.

[10] Bram A.D., Gottschalk K.A., Leeds W.M. (2018). Emotional Regulation in Women with Chronic Fatigue Syndrome and Depression: Internal Representations and Adaptive Defenses. J. Am. Psychoanal. Assoc..

[11] Manning K., Bakhshaie J., Shepherd J.M., Jones J., Timpano K.R., Viana A.G., Zvolensky M.J. (2019). Fatigue severity and emotion dysregulation: Roles in mental health among trauma exposed college students. Fatigue Biomed. Heal. Behav..

[12] Lewczuk K., Wizła M., Oleksy T., Wyczesany M. (2022). Emotion Regulation, Effort and Fatigue: Complex Issues Worth Investigating. Front. Psychol..

[13] Edwards R., Suresh R., Lynch S., Clarkson P., Stanley P. (2001). Illness perceptions and mood in chronic fatigue syndrome. J. Psychosom. Res..

[14] Rakib A., White P.D., Pinching A.J., Hedge B., Newbery N., Fakhoury W.K., Priebe S. (2005). Subjective quality of life in patients with chronic fatigue syndrome. Qual. Life Res..

[15] Haines C., Loades M., Davis C. (2018). Illness perceptions in adolescents with chronic fatigue syndrome and other physical health conditions: Application of the common sense model. Clin. Child Psychol. Psychiatry.

[16] Roberts D. (2018). Chronic fatigue syndrome and quality of life. Patient Relat. Outcome Meas..

[17] de Boer A.G.E.M., de Wind A., Coenen P., van Ommen F., Greidanus M.A., Zegers A.D., Duijts S.F.A., Tamminga S.J. (2023). Cancer survivors and adverse work outcomes: Associated factors and supportive interventions. Br. Med. Bull..

[18] Macfarlane G.J., D’angelo S., Ntani G., Walker-Bone K. (2024). Impact of fatigue on work productivity and health-related job loss. Occup. Med..

[19] Sosso F.A.E., Silva F.T., Rodrigues R.Q., Carvalho M.M., Zoukal S., Zarate G.C. (2023). Prevalence of Sleep Disturbances in Latin American Populations and Its Association with Their Socioeconomic Status—A Systematic Review and a Meta-Analysis. J. Clin. Med..

[20] Valero S., Sáez-Francàs N., Calvo N., Alegre J., Casas M. (2013). The role of neuroticism, perfectionism and depression in chronic fatigue syndrome. A structural equation modeling approach. Compr. Psychiatry.

[21] Wright A., Fisher P.L., Baker N., O’Rourke L., Cherry M.G. (2021). Perfectionism, depression and anxiety in chronic fatigue syndrome: A systematic review. J. Psychosom. Res..

[22] Costa A.P., Brito I.d.S., Mestre T.D., Pires A.M., Lopes M.J. (2025). Meshing Anxiety, Depression, Quality of Life, and Functionality in Chronic Disease. Healthcare.

[23] Wyller V.B., Eriksen H.R., Malterud K. (2009). Can sustained arousal explain the Chronic Fatigue Syndrome?. Behav. Brain Funct..

[24] Bruno A., Rizzo A., Muscatello M.R.A., Celebre L., Silvestri M.C., Zoccali R.A., Mento C. (2020). Hyperarousal Scale: Italian Cultural Validation, Age and Gender Differences in a Nonclinical Population. Int. J. Environ. Res. Public Health.

[25] Liu C., Rotaru K., Lee R.S.C., Tiego J., Suo C., Yücel M., Albertella L. (2021). Distress-driven impulsivity interacts with cognitive inflexibility to determine addiction-like eating. J. Behav. Addict..

[26] Su Y., Cochrane B.B., Reding K., Herting J.R., Tinker L.F., Zaslavsky O. (2022). Mediterranean Diet and Fatigue among Community-Dwelling Postmenopausal Women. J. Nutr. Gerontol. Geriatr..

[27] Arslan S., Bozkurt C., Arslan M., Bulut H. (2023). Effects of adherence to the Mediterranean diet on fatigue and activities of daily living in geriatric individuals with COPD. Clin. Nutr. ESPEN.

[28] American Academy of Sleep Medicine (AASM) International Classification of Sleep Disorders, Third Edition, Text Revision (ICSD-3-TR). https://aasm.org/clinical-resources/international-classification-sleep-disorders/.

[29] Vignatelli L., Plazzi G., Barbato A., Ferini-Strambi L., Manni R., Pompei F., D’Alessandro R. (2003). Italian version of the Epworth sleepiness scale: External validity. Neurol. Sci..

[30] Krupp L.B., LaRocca N.G., Muir-Nash J., Steinberg A.D. (1989). The fatigue severity scale. Application to patients with multiple sclerosis and systemic lupus erythematosus. Arch. Neurol..

[31] Buysse D.J., Reynolds C.F., Monk T.H., Berman S.R., Kupfer D.J. (1989). The Pittsburgh sleep quality index: A new instrument for psychiatric practice and research. Psychiatry Res..

[32] Hallion L.S., Steinman S.A., Tolin D.F., Diefenbach G.J. (2018). Psychometric Properties of the Difficulties in Emotion Regulation Scale (DERS) and Its Short Forms in Adults With Emotional Disorders. Front. Psychol..

[33] Bottesi G., Ghisi M., Altoè G., Conforti E., Melli G., Sica C. (2015). The Italian version of the Depression Anxiety Stress Scales-21: Factor structure and psychometric properties on community and clinical samples. Compr. Psychiatry.

[34] Rossi A.A., Mannarini S., Castelnuovo G., Pietrabissa G. (2022). Disordered Eating Behaviors Related to Food Addiction/Eating Addiction in Inpatients with Obesity and the General Population: The Italian Version of the Addiction-like Eating Behaviors Scale (AEBS-IT). Nutrients.

[35] Zhang W., Bansback N., Boonen A., Young A., Singh A., Anis A.H. (2010). Validity of the work productivity and activity impairment questionnaire-general health version in patients with rheumatoid arthritis. Arthritis Res. Ther..

[36] Sofi F., Macchi C., Abbate R., Gensini G.F., Casini A. (2014). Mediterranean diet and health status: An updated meta-analysis and a proposal for a literature-based adherence score. Public Health Nutr.

[37] Brooks R., Boye K.S., Slaap B. (2020). EQ-5D: A plea for accurate nomenclature. J. Patient-Rep. Outcomes.

[38] The Jamovi Project, Jamovi. (Version 2.3) [Computer Software]. http://www.jamovi.org.

[39] Douglas C.E., Michael F.A. (1991). On distribution-free multiple comparisons in the one-way analysis of variance. Commun. Stat.-Theory Methods.

[40] Hollander M., Wolfe D.A., Chicken E. (2015). Nonparametric Statistical Methods.

[41] Ramos J.N., Muraro A.P., Nogueira P.S., Ferreira M.G., Rodrigues P.R.M. (2021). Poor sleep quality, excessive daytime sleepiness and association with mental health in college students. Ann. Hum. Biol..

[42] Tsou M.-T., Chang B.C.-C. (2019). Association of Depression and Excessive Daytime Sleepiness among Sleep-Deprived College Freshmen in Northern Taiwan. Int. J. Environ. Res. Public Health.

[43] Schneider L.D., Stevens J., Husain A.M., Ito D., Fuller D.S., Zee P.C., Macfadden W. (2023). Symptom Severity and Treatment Satisfaction in Patients with Idiopathic Hypersomnia: The Real World Idiopathic Hypersomnia Outcomes Study (ARISE). Nat. Sci. Sleep.

[44] Léger D., Stepnowsky C. (2020). The economic and societal burden of excessive daytime sleepiness in patients with obstructive sleep apnea. Sleep Med. Rev..

[45] Waldman L.T., Parthasarathy S., Villa K.F., Bron M., Bujanover S., Brod M. (2020). Understanding the burden of illness of excessive daytime sleepiness associated with obstructive sleep apnea: A qualitative study. Health Qual. Life Outcomes.

[46] Younossi Z.M., Kremer A.E., Swain M.G., Jones D., Bowlus C., Trauner M., Henry L., Gerber L. (2024). Assessment of fatigue and its impact in chronic liver disease. J. Hepatol..

[47] Torlinska B., Raza K., Filer A., Jutley G., Sahbudin I., Singh R., de Pablo P., Rankin E., Rhodes B., Amft N. (2024). Predictors of quality of life, functional status, depression and fatigue in early arthritis: Comparison between clinically suspect arthralgia, unclassified arthritis and rheumatoid arthritis. BMC Musculoskelet. Disord..

[48] Eppingbroek A.A.M., Lechner L., Bakker E.C., Nijkamp M.D., de Witte M.A., Bolman C.A.W. (2024). The personal impact of living with a myeloproliferative neoplasm. Psycho-Oncology.

[49] Ramirez A.O., Keenan A., Kalau O., Worthington E., Cohen L., Singh S. (2021). Prevalence and burden of multiple sclerosis-related fatigue: A systematic literature review. BMC Neurol..

[50] Patwardhan A., Kamble N., Bhattacharya A., Holla V., Yadav R., Pal P.K. (2024). Impact of Non-Motor Symptoms on Quality of Life in Patients with Early-Onset Parkinson’s Disease. Can. J. Neurol. Sci./J. Can. des Sci. Neurol..

[51] Huang L., Zhu W., Li N., Zhang B., Dai W., Li S., Xu H. (2024). Functions and mechanisms of adenosine and its receptors in sleep regulation. Sleep Med..

[52] Kampaite A., Gustafsson R., York E.N., Foley P., MacDougall N.J.J., Bastin M.E., Chandran S., Waldman A.D., Meijboom R. (2024). Brain connectivity changes underlying depression and fatigue in relapsing-remitting multiple sclerosis: A systematic review. PLoS ONE.

[53] Filippi M., Rocca M., Colombo B., Falini A., Codella M., Scotti G., Comi G. (2002). Functional Magnetic Resonance Imaging Correlates of Fatigue in Multiple Sclerosis. NeuroImage.

[54] Wilting J., Rolfsnes H.O., Zimmermann H., Behrens M., Fleischer V., Zipp F., Gröger A. (2015). Structural correlates for fatigue in early relapsing remitting multiple sclerosis. Eur. Radiol..

[55] Penner I., Raselli C., Stöcklin M., Opwis K., Kappos L., Calabrese P. (2009). The Fatigue Scale for Motor and Cognitive Functions (FSMC): Validation of a new instrument to assess multiple sclerosis-related fatigue. Mult. Scler. J..

[56] Vaccarino V., Bremner J.D. (2017). Behavioral, emotional and neurobiological determinants of coronary heart disease risk in women. Neurosci. Biobehav. Rev..

[57] Farrelly C. (2023). Longevity Science and Women’s Health and Wellbeing. J. Popul. Ageing.

[58] World Health Organization (2025). World Health Observatory: Healthy Life Expectancy (HALE) at Birth (Years). https://www.who.int/data/gho/data/indicators/indicator-details/GHO/gho-ghe-hale-healthy-life-expectancy-at-birth.

[59] Nusselder W.J., Cambois E.M., Wapperom D., Meslé F., Looman C.W.N., Yokota R.T.C., Van Oyen H., Jagger C., Robine J.M. (2019). Women’s excess unhealthy life years: Disentangling the unhealthy life years gap. Eur. J. Public Health.

[60] Castelletti G., Misirocchi F., Zilioli A., Salvatelli M.L., Rausa F., Pizzarotti S., Zinno L., Florindo I., Pedrazzi G., Parrino L. (2023). How can sleep disorders affect our reaction towards external stressors: A lesson from the COVID-19 outbreak. Neurol. Sci..

[61] Riemann D., Spiegelhalder K., Feige B., Voderholzer U., Berger M., Perlis M., Nissen C. (2010). The hyperarousal model of insomnia: A review of the concept and its evidence. Sleep Med. Rev..

[62] Belloir J., Makarem N., Shechter A. (2022). Sleep and Circadian Disturbance in Cardiovascular Risk. Curr. Cardiol. Rep..

[63] Lim V.K., Chen D., Aw S.S., Tan M. (2016). Unemployed and exhausted? Job-search fatigue and reemployment quality. J. Vocat. Behav..

[64] Blanchflower D.G., Bryson A. (2021). Unemployment and sleep: Evidence from the United States and Europe. Econ. Hum. Biol..

[65] Torquati L., Mielke G.I., Brown W.J., Burton N.W., Kolbe-Alexander T.L. (2019). Shift Work and Poor Mental Health: A Meta-Analysis of Longitudinal Studies. Am. J. Public Health.

[66] Morgan T., Basalely A., Singer P., Castellanos L., Sethna C.B. (2024). The association between sleep duration and cardiometabolic risk among children and adolescents in the United States (US): A NHANES study. Child Care Health Dev..

[67] Chaput J.-P., Dutil C., Featherstone R., Ross R., Giangregorio L., Saunders T.J., Janssen I., Poitras V.J., Kho M.E., Ross-White A. (2020). Sleep duration and health in adults: An overview of systematic reviews. Appl. Physiol. Nutr. Metab..

[68] Arab A., Karimi E., Garaulet M., Scheer F.A. (2024). Dietary patterns and insomnia symptoms: A systematic review and meta-analysis. Sleep Med. Rev..

