# Supplementary material for: The Overlapping Burdens of Fatigue and Daytime Sleepiness: Gender-Specific Impacts on Life Quality in Patients with Sleep Disorders
Source: Diseases. 2025 May 29;13(6):172. doi: 10.3390/diseases13060172 (PMC12191932; doi:10.3390/diseases13060172)
Supplement: Supplementary file 1 [file diseases-13-00172-s001.zip › diseases-3640764-supplementary.pdf]

The overlapping burdens of fatigue and daytime sleepiness: gender-specific impacts on life quality in patients with sleep disorders

Supplementary Materials

	Insomnia	SRBD	CDH	CSWD	SRMD	Parasomnia	SRE
**N (%)**	52 (38.2)	47 (34.6)	15 (11)	3 (2.2)	8 (5.9)	8 (5.9)	3 (2.2%)
**BMI, mean (SD)**	24.6 (4.91)	30.1 (4.67)	25.5 (3.63)	25.2 (3.02)	26.9 (6.92)	25.1 (3.66)	20.5 (2.14)
**ESS, mean (SD)**	5.75 (4.42)	7.64 (4.74)	11.9 (4.67)	8.67 (5.13)	10.5 (3.51)	7.63 (3.50)	7.67 (8.02)
**FSS, mean (SD)**	37.3 (17.2)	30.9 (15)	30.9 (19.3)	29.7 (13.7)	40.9 (13.3)	27.4 (16.3)	26 (17.5)
**PSQI mean (SD)**	11.4 (4.03)	7.70 (4.12)	8.67 (4.58)	8 (2)	12 (4.14)	6.88 (4.29)	8.33 (6.11)
**DERS mean (SD)**	42.7 (10.1)	39.9 (11.6)	38 (10.7)	51.3 (14.6)	42.3 (14.2)	42.1 (14.6)	28.3 (15.4)
**AES**	38.4 (6.26)	38.9 (9.03)	37.3 (8.45)	43.7 (13.6)	38.6 (5.83)	34.9 (5.54)	33.7 (3.21)
**H-scale**	31 (13.9)	23.1 (12.1)	21.7 (11.8)	28.3 (24.1)	24.3 (12.3)	31 (17.8)	16.07 (2.08)
**EQ-5D**	4.25 (2.57)	3.36 (2.29)	4.20 (2.04)	2 (0)	3.75 (1.67)	2.63 (2.33)	2.67 (3.06)
**DASS-depression**	29.1 (18.7)	21.4 (19.1)	22.1 (21.7)	46.7 (24.1)	22 (10.3)	29.5 (31.1)	13.3 (23.1)
**DASS-anxiety**	19.4 (14.4)	17.1 (13.7)	17.3 (19)	26.7 (22.7)	26.5 (18.3)	18 (26.3)	12 (20.8)
**DASS-stress**	38.9 (18.3)	24.9 (18.5)	24.9 (15.1)	49.3 (12.9)	25.5 (14)	28 (22.9)	20 (17.4)
**MEDI-Lite**	8.90 (2.71)	8.19 (2.34)	8.87 (3)	7 (0)	8.63 (2.72)	9.88 (3.18)	9.67 (4.04)
**WPAI: work time % missed due to health**	0.04 (0.17)	0.06 (0.20)	0.05 (0.17)	0 (0)	0.01 (0.01)	0.08 (0.20)	0 (0)
**WPAI impairment while working due to health**	0.32 (0.23)	0.31 (0.25)	0.32 (0.25)	0.33 (0.25)	0.43 (0.25)	0.30 (0.25)	0.15 (0.07)
**WPAI overall work impairment due to heath**	0.32 (0.24)	0.30 (026)	0.33 (0.29)	0.33 (0.25)	0.43 (0.25)	0.35 (0.29)	0.15 (0.07)
**WPAI activity impairment due to health**	0.42 (0.29)	0.33 (0.26)	0.42 (0.34)	0.36 (0.25)	0.48 (0.28)	0.32 (0.29)	0.23 (0.25)

Table S1: *Questionnaires results according to sleep disorder diagnosis. SRBD sleep-related breathing disorders, CDH central disorders of hypersomnolence, CSWD circadian sleep-wake disorders, SRMD sleep-related movement disorders, SRE sleep-related epilepsy, BMI body mass index, ESS Epworth Sleepiness Scale, FSS fatigue severity scale, PSQI Pittsburgh Sleep Quality Index, DERS Difficulties in Emotion Regulation Scale, AES Addiction-like eating behaviour scale, H-scale hyperarousal scale, EQ-5D, DASS-21 Depression-Anxiety Stress Scale-21, WPAI work productivity and activity impairment.*

	Female	Male	p	Cohen's d
N	60	76		
Age	53.2 (13.7)	51.4 (13.2)	0.434	0.078
BMI	25.9 (6.10)	27.6 (4.68)	0.069	0.199
ESS	8.37 (4.99)	6.95 (4.72)	0.094	0.891
FSS	41.2 (16.4)	27.6 (14)	<0.001*	0.891
PSQI	10.78 (4.23)	8.38 (4.39)	0.002*	0.557
DERS	44.23 (12.18)	38.46 (10.37)	0.004*	0.510
EBS	39.55 (7.45)	37.28 (7.68)	0.084	0.300
H-scale	32.45 (15.33)	21.75 (10.15)	< 0.001*	0.823
EQ-5D	4.55 (2.31)	3.08 (2.23)	<0.001*	0.649
DASS-depression	32.33 (21.16)	19.76 (17.48)	<.0001*	0.648
DASS-anxiety	24.80 (16.94)	13.92 (13.43)	<.0001*	0.712
DASS-stress	36.93 (18.12)	25.55 (18.66)	<.0001*	0.619
MEDI-LITE	9.20 (2.60)	8.25 (2.62)	0.037*	0.364
WPAI: work time % missed due to health	0.08 (0.187)	0.03 (0.144)	0.151	0.329
WPAI impairment while working due to health	0.40 (0.249)	0.26 (0.212)	0.013*	0.572
WPAI overall work impairment due to heath	0.42 (0.265)	0.26 (0.227)	0.007*	0.632
WPAI activity impairment due to health	0.47 (0.308)	0.31 (0.249)	0.001*	0.585

Table S2: *Questionnaires results according to gender-related differences. BMI body mass index, ESS Epworth Sleepiness Scale, FSS fatigue severity scale, PSQI Pittsburgh Sleep Quality Index, DERS Difficulties in Emotion Regulation Scale, AES Addiction-like eating behaviour scale, H-scale hyperarousal scale, EQ-5D, DASS-21 Depression-Anxiety Stress Scale-21, WPAI work productivity and activity impairment.*

	Employed	Unemployed	Retired	p-value	E2
N	90	10	36		
Age	47.6	46.6	65.4*	<.001*	0.431
BMI	26.8 (5.35)	24.2 (5.98)	28 (5.04)	0.194	0.028
ESS	7.82 (5.28)	8.10 (5.69)	6.81 (3.44)	0.703	0.005
FSS	31.2 (5.28)	33.8 (15.8)	54.6 (6.65)	<0.001*	0.131
PSQI	9.83 (4.42)	11.7 (4.67)	7.83 (4.16)	0.025*	0.054
DERS	40.9 (11.5)	48.3 (12.1)	39.3 (10.9)	0.115	0.032
EBS	38.4 (7.80)	40.4 (9.11)	37.4 (6.84)	0.471	0.011
H-scale	26.5 (13.4)	35.2 (15)	24.1 (13.6)	0.072	0.039
EQ-5D	3.64 (2.52)	5.20 (1.93)	3.53 (1.98)	0.093	0.035
DASS-depression	24.6 (20.5)	35.2 (16.6)	24.2 (19.8)	0.167	0.026

**DASS-anxiety**	17.7 (16.3)	30.4 (17.9)	18.1 (13.6)	0.076	0.038
**DASS-stress**	32 (19.7)	32.4 (15.4)	26.4 (18.7)	0.420	0.017
**MEDI-LITE**	8.57 (2.90)	9.80 (2.49)	8.61 (1.92)	0.311	0.017

Table S3: *Questionnaires results according to occupational status. BMI body mass index, ESS Epworth Sleepiness Scale, FSS fatigue severity scale, PSQI Pittsburgh Sleep Quality Index, DERS Difficulties in Emotion Regulation Scale, AES Addiction-like eating behaviour scale, H-scale hyperarousal scale, EQ-5D, DASS-21 Depression-Anxiety Stress Scale-21.*

	**Shift-workers**	**Non-shift workers**	**p-value**	**Cohens' d**
**N**	29	107		
**Age**	44.4 (12.5)	54.3 (12.8)	<.001*	0.785
**BMI**	26.7 (5.07)	27 (5.46)	0.843	0.046
**ESS**	7.31 (5.29)	7.64 (4.78)	0.759	0.066
**FSS**	33 (16.38)	33.74 (16.59)	0.831	0.044
**PSQI**	10.93 (4.88)	9.04 (4.28)	0.065	-0.411
**DERS**	44 (10.96)	40.20 (11.59)	0.108	-0.337
**EBS**	39.21 (7.87)	38.03 (7.59)	0.475	-0.152
**H-scale**	26.24 (14.37)	26.53 (13.61)	0.922	0.020
**EQ-5D**	3.79 (2.69)	3.71 (2.29)	0.880	-0.033
**DASS-depression**	27.03 (22.97)	24.84 (19.37)	0.640	-0.103
**DASS-anxiety**	20.97 (19.32)	18.11 (14.98)	0.465	-0.165
**DASS-stress**	33.38 (18.98)	29.81 (19.29)	0.376	-0.186
**MEDI-LITE**	7.79 (2.14)	8.91 (2.72)	0.023*	0.454
**WPAI: work time % missed due to health**	0.04 (0.160)	0.05 (0.165)	0.735	0.078
**WPAI impairment while working due to health**	0.34 (0.271)	0.30 (0.218)	0.488	-0.168
**WPAI overall work impairment due to heath**	0.30 (0.244)	0.33 (0.258)	0.617	0.117
**WPAI activity impairment due to health**	0.43 (0.30)	0.37 (0.284)	0.335	-0.207

Table S4: *Questionnaires results according to shift-working. BMI body mass index, ESS Epworth Sleepiness Scale, FSS fatigue severity scale, PSQI Pittsburgh Sleep Quality Index, DERS Difficulties in Emotion Regulation Scale, AES Addiction-like eating behaviour scale, H-scale hyperarousal scale, EQ-5D, DASS-21 Depression-Anxiety Stress Scale-21, WPAI work productivity and activity impairment.*

	FSS	ESS
**PSQI**	Spearman's Rho 0.365<br>p-value <0.001*	Spearman's Rho -0.028<br>p-value 0.748
**DERS**	Spearman's Rho 0.519<br>p-value <0.001*	Spearman's Rho r 0.078<br>p-value 0.368
**EBS**	Spearman's Rho 0.328<br>p-value <0.001*	Spearman's Rho 0.152<br>p-value 0.077
**H-scale**	Spearman's Rho 0.297<br>p-value < 0.001*	Spearman's Rho 0.118<br>p-value 0.173
**EQ-5D**	Spearman's Rho 0.552<br>p-value <0.001*	Spearman's Rho 0.045<br>p-value 0.604
**DASS-depression**	Spearman's Rho 0.466<br>p-value<0.001*	Spearman's Rho 0.110<br>p-value 0.204
**DASS-anxiety**	Spearman's Rho 0.505<br>p-value < 0.001*	Spearman's Rho 0.146<br>p-value 0.089
**DASS-stress**	Spearman's Rho 0.421<br>p-value <.0001*	Spearman's Rho 0.063<br>p-value 0.466
**MEDI-LITE**	Spearman's Rho -0.062<br>p-value 0.474	Spearman's Rho 0.012<br>p-value 0.885
**WPAI: work time % missed due to health**	Spearman's Rho 0.278<br>p-value 0.009*	Spearman's Rho 0.230<br>p-value 0.032*
**WPAI impairment while working due to health**	Spearman's Rho 0.442<br>p-value <0.001*	Spearman's Rho 0.230<br>p-value 0.032*
**WPAI overall work impairment due to heath**	Spearman's Rho 0.446<br>p-value <0.001*	Spearman's Rho 0.301<br>p-value 0.005*
**WPAI activity impairment due to health**	Spearman's Rho 0.620<br>p-value <0.001*	Spearman's Rho 0.183<br>p-value 0.033*

Table S5: *Correlation analysis between FSS and ESS and main questionnaires' results. BMI body mass index, ESS Epworth Sleepiness Scale, FSS fatigue severity scale, PSQI Pittsburgh Sleep Quality Index, DERS Difficulties in Emotion Regulation Scale, AES Addiction-like eating behaviour scale, H-scale hyperarousal scale, EQ-5D, DASS-21 Depression-Anxiety Stress Scale-21, WPAI work productivity and activity impairment.*
